# Role for the metalloproteinase ADAM28 in the control of airway inflammation, remodelling and responsiveness in asthma

**DOI:** 10.3389/fimmu.2022.1067779

**Published:** 2023-01-05

**Authors:** Guillaume Bendavid, Céline Hubeau, Fabienne Perin, Alison Gillard, Marie-Julie Nokin, Oriane Carnet, Catherine Gerard, Agnès Noel, Philippe Lefebvre, Natacha Rocks, Didier Cataldo

**Affiliations:** ^1^ Laboratory of Tumor and Development Biology, GIGA-Cancer, University of Liege (ULiege), Liege, Belgium; ^2^ Department of Otorhinolaryngology Head and Neck Surgery, University of Liege (ULiege) and Centre Hospitalier Universitaire (CHU) Liege, Liege, Belgium; ^3^ Department of respiratory diseases, University of Liege (ULiege) and Centre Hospitalier Universitaire (CHU) Liege, Liege, Belgium

**Keywords:** asthma, proteases, adamalysins, ADAM28, mouse model, airway remodelling

## Abstract

**Background:**

Asthma is characterized by morphological modifications of the airways (inflammation and remodelling) and bronchial hyperresponsiveness. Mechanisms linking these two key features of asthma are still poorly understood. ADAM28 (a disintegrin and metalloproteinase 28) might play a role in asthma pathophysiology. ADAM28 exists as membrane-bound and soluble forms and is mainly expressed by lymphocytes and epithelial cells.

**Methods:**

ADAM28^-/-^ mice and ADAM28^+/+^ counterparts were sensitized and exposed to ovalbumin (OVA). Airway responsiveness was measured using the flexiVent^®^ system. After sacrifice, bronchoalveolar lavage (BAL) was performed and lungs were collected for analysis of airway inflammation and remodelling.

**Results:**

The expression of the soluble form of ADAM28 was lower in the lungs of OVA-exposed mice (as compared to PBS-exposed mice) and progressively increased in correlation with the duration of allergen exposure. In lungs of ADAM28^-/-^ mice exposed to allergens, the proportion of Th2 cells among ${\text{CD}}_{4}^{+}$ cells and the number of B cells were decreased. Bronchial responsiveness was lower in ADAM28^-/-^ mice exposed to allergens and similar to the responsiveness of sham-challenged mice. Similarly, features of airway remodelling (collagen deposition, smooth muscle hyperplasia, mucous hyperplasia) were significantly less developed in OVA-exposed ADAM28^-/-^ animals in sharp contrasts to ADAM28^+/+^. In addition, we report the first evidence of ADAM28 RNA expression by lung fibroblasts and we unveil a decreased capacity of lung fibroblasts extracted from OVA-exposed ADAM28^-/-^ mice to proliferate as compared to those extracted from OVA-exposed ADAM28^+/+^ suggesting a direct contribution of this enzyme to the modulation of airway remodelling.

**Conclusion:**

These results suggest that ADAM28 might be a key contributor to the pathophysiology of asthma.

## Background

Asthma is an inflammatory disease of the airways of increasing prevalence worldwide (1). The vast majority of asthmatics see their disease adequately controlled with currently available standard therapies and a minority of severe asthmatics remains uncontrolled and requires targeted treatments with biologicals aiming at interfering with the disease process (1). With an impaired quality of life and a number of disease-related complications, this subgroup of patients is responsible for the majority of asthma-related costs (healthcare resources, drugs, hospitalizations, working day loss, etc). Moreover, asthmatics displaying an accelerated decline of lung function during their lifetime or a fixed airway obstruction display significantly more profound airway remodelling (2, 3). Airway remodelling in asthma is a characteristic of the disease and includes Goblet cell hyperplasia, basement membrane thickening, collagen deposition around the airways as well as smooth muscle hyperplasia (4). The biological mechanisms leading to an established airway remodelling are still not fully unveiled but there is a strong influence of airway epithelial cells. Indeed, the airway epithelial cells collected from asthmatic donors display an enhanced expression of remodelling- related genes (5, 6). These characteristics of the asthmatic airway epithelial cells affect the profibrogenic potential of the airway fibroblasts that contribute to profoundly modify the extracellular matrix of the bronchi (7, 8). Although many different pathways might contribute to the activation of fibroblasts, transforming growth factor (TGF)-β induced fibroblast activation that triggers extracellular matrix production is key (9). The biology of TGF- β is complex since this mediator requires a post-translational activation that can be achieved by different mechanisms including a cleavage by matrix metalloproteinases (MMPs) (10).

A disintegrin and metalloproteinases (ADAMs) are membrane-bound or secreted enzymes that display the characteristics to play an important role in the regulation of inflammation and remodelling since they are able to process many soluble or membrane-bound mediators (including a number of chemokines/cytokines), hence modifying their biological activity (11). The prototypical example is the membrane-bound pro-TNF-alpha that requires a cleavage by ADAM proteases before being activated and released in a soluble form (11, 12). Some of these enzymes also cleave different components of the extracellular matrix. The ADAM proteins are matrix metalloproteinase (MMPs)-related enzymes, bearing a multi-domain structure. They have been associated to numerous physiological and pathological processes to date (11). Modulation of the expression of different ADAM(TS) in the bronchial tree of a cohort of human asthmatics has been reported (13, 14).

ADAM28 is a multipotent membrane-bound proteinase expressed by tissues derived from the foregut in embryo suggesting its involvement in respiratory tract organogenesis. Membrane-bound ADAM28 can be released after proteolytic cleavage by different proteinases including MMP7 resulting in the release of a soluble form of ADAM28 as it has been also shown for ADAM33 (15, 16). Interestingly, the soluble form of ADAM28 has been reported to enhance the α4β1-dependent cell adhesion to vascular cell adhesion molecule-1 (VCAM-1) and therefore it influences lymphocyte adhesion and trans-endothelial migration (17). Various ADAM28 splicing variants have been described. Notably, microarray studies show an upregulation of ADAM28 expression after induction of lung inflammation in a mouse model of chronic asthma (18) and ADAM28 is expressed by airway epithelial cells (19). Moreover, ADAM28 deficiency has been associated with impaired ${\text{CD}}_{8}^{+}$ T recruitment in lung and spleen contributing to a protective role for host ADAM28 against metastasis dissemination of cancer cells (20). This protease can therefore play different roles in the microenvironment either in its soluble or membrane-bound form.

In this article, we establish a strong relationship between the expression of ADAM28 and asthma-associated bronchial hyperresponsiveness and remodelling. ADAM28 deficient mice display lower infiltration of bronchial walls and bronchoalveolar lavage (BAL) by inflammatory cells, reduced features of airway remodelling, and lower hyperresponsiveness as compared to wild-type mice.

## Materials and methods

### Sensitization followed by allergen sensibilisation

**Figure 1:**
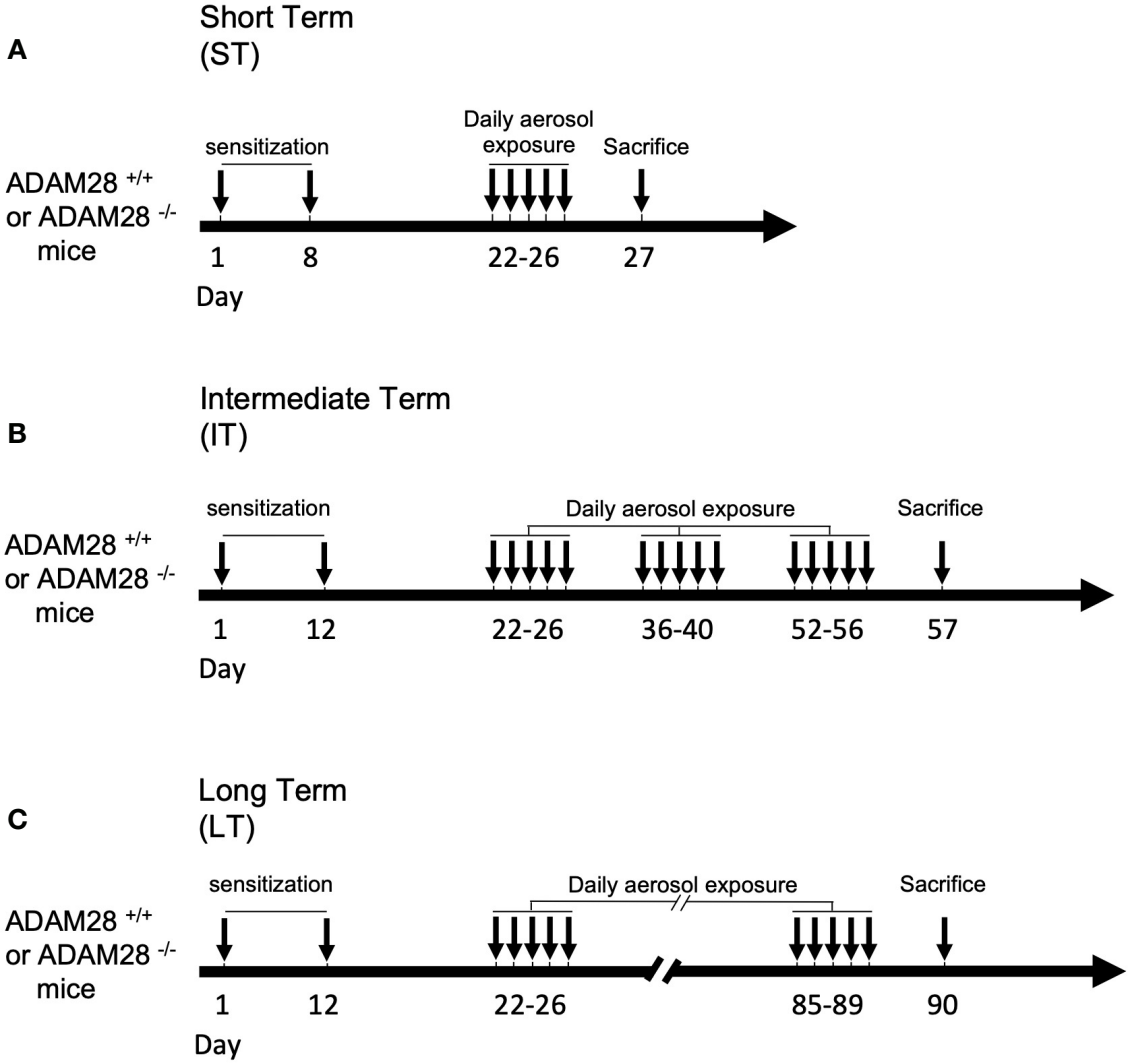
Experimental procedure. Short-term (ST) protocol **(A)**, intermediate-term (IT) protocol **(B)**, and long-term (LT) protocol **(C)**. PBS/ovalbumin (OVA) sensitization and exposure protocols. Mice were sensitized on days 1 and 8 by intraperitoneal injection of 10 μg of OVA. Mice were subsequently exposed to daily PBS aerosol or OVA 1% aerosol for 30 min for ST, IT, or LT duration (see materials and methods section for the detailed protocol).

Full Knock out mice (ADAM28^-/-^) for ADAM28 BALB/c mice and Wild type (ADAM28^+/+^) counterparts were previously described (20) and were enrolled in our studies according to “Principles of Laboratory Animal Care” (National Society for Medical Research). Experimental protocols described were approved by the animal ethical committee of the University of Liège (under the references #1597 and #2146). All experiments were performed on male and female mice. Animals were included in the asthma protocol aged 8 weeks and their weight was between 20 and 25 grams. Three protocols of asthma induction were used and referred to as a “short-term” (ST), “intermediate-term” (IT) and a “long-term” (LT) exposure protocol (**Figure 1**). Eight weeks-old BALB/c mice were sensitized on days 1 and 8 (ST model) or on days 1 and 12 (IT and LT models) by intraperitoneal injection of 10 μg of ovalbumin (OVA Grade V; ref#A5503 Sigma-Aldrich, Schnelldorf, Germany) emulsified in 2 mg aluminium hydroxide (AlumInject; ref#77161 Perbio, Erembodegem, Belgium). Mice were subsequently divided into 4 groups for daily nebulization in standard Plexiglas boxes (30 × 20 × 15 cm): 2 groups of mice (ADAM28^-/-^ and ADAM28^+/+^ mice) were only exposed to PBS (ref#17-516Q, Lonza, Verviers, Belgium) aerosol (control cohorts), and the 2 other groups (ADAM28^-/-^ mice and ADAM28^+/+^) were subjected to ovalbumin 1% aerosol for 30 min (OVA Grade III; ref#A5378 Sigma-Aldrich, Schnelldorf, Germany). Aerosols were generated daily by ultrasonic nebulizer from days 22 to 26 for the ST exposure model and from days 22 to 56 for the IT model (aerosols were continued until day 90 for the LT model) 5 days/week (every odd week).

### Assessment of airway responsiveness

Mice were exposed to the 90 days model of exposition to OVA. At the day of sacrifice, they were anesthetized by intraperitoneal injection of pentobarbital (70 mg/kg, Nembutal^®^, Sanofi Animal Health, Belgium) and ketamin (75 mg/kg, Ketalar^®^, Pfizer, Brussels, Belgium). A surgical tracheotomy was performed and was followed by insertion of an 18-gauge polyethylene catheter into the trachea. Mice were ventilated by using a FlexiVent small animal ventilator (SCIREQ, Montréal, Québec, Canada). Baseline lung-function was evaluated during FlexiVent manoeuvres measuring lung compliance, tissue elastance, and tissue hysteresivity in basal conditions. Lung hysteresis, the area between the ascending and descending portions of the pressure-volume curve is a reflect of the property of the lung to dissipate the received energy (the energy applied to the lung in inspiration is not completely recovered in expiration). Hysteresivity (η) is the ratio of tissue damping (G) over tissue elastance (H). After basal evaluation, mice were exposed by inhalation to increasing doses of nebulized methacholine (1.25, 2.5, 5, 10, 20 mg/ml; ref#190231 ICN, Asse-Relegem, Belgium, PBS was used as a diluting solvent), and a dose-response (airway resistance) curve was obtained for each animal.

### Measurement of airway remodelling and airway inflammation

Right main bronchus was clamped and right lung was excised and immediately frozen in liquid nitrogen. The remaining left lung was inflated with 4% paraformaldehyde (PFA, Ref#8.18715.1000, Sigma-Aldrich, Schnelldorf, Germany) and resected. After a night in PFA bath, lungs were embedded in paraffin. Five-µm sections were stained with haematoxylin–eosin. A peribronchial inflammation score was determined, related to cell infiltration around the bronchi, by quantification of peribronchial inflammatory cells (eosinophils, lymphocytes, macrophages, etc.), as previously described (21). When no inflammatory cells were detectable around the bronchi a value of 0 was given. A value of 1 was given when there were occasionally inflammatory cells, a value of 2 when most bronchi were surrounded by a thin layer (one to five cells) of inflammatory cells, and a value of 3 when most bronchi were surrounded by a thick layer (more than five cells) of inflammatory cells. The score was measured on seven randomly selected bronchial sections per mouse and peribronchial inflammation scores are expressed as a mean value per animal.

Masson’s Trichrome staining was used to measure collagen deposition around the bronchi. As previously described, a collagen deposition score was applied (allowing to give a score from 0 to 3 to each observed bronchi) (18). A score of 0 was recorded when no collagen was stained by Trichrome Masson around the bronchi, a score of 1 for a thin layer of collagen, 2, for a cluster of collagens and 3, for a thick layer of collagen. Immunohistochemistry for alpha-smooth muscle actin (α-SMA) was performed using mouse primary antibody anti-α-SMA-FITC (ref#F3777 Sigma–Aldrich, Schnelldorf, Germany). Digitalized slides corresponding to 7 bronchi per mice were analysed. Smooth-muscle-cell layer was measured and reported to epithelial basement membrane perimeter. Glandular hyperplasia was evaluated by measuring the percentage of periodic acid-Schiff (PAS)-stained goblet cells per total epithelial cells (percentage of 300 randomly counted cells) (PAS ref#1.09034.1000, Merck, Hoeilaart, Belgium).

### Lung fibroblasts: Culture, proliferation test and RNA analyse

Fibroblasts were isolated from the lung of ADAM28^+/+^ mice exposed or non-exposed to allergens in LT model of asthma. They were cultured according to already validated protocols (22). Immediately after sacrifice, chests of mice were disinfected with 70% ethanol and lungs were excised using sterile tools. They were cut into small pieces by cutting with razor blades. Tissue digestion was performed by collagenase (Collagenase from Clostridium histolyticum, ref#C9891 Sigma-Aldrich). Fibroblasts were cultured in hypoxia (incubator at 37°C, 5% of CO2 and 3% of O2). Confluence of the fibroblasts was followed using microscope. When they covered 60% of the plate medium was changed to remove debris and unattached cells. Between days 7 and 14, according to fibroblasts confluence, cells were harvested and divided in several plates for analyse. One of them was reserved for further RNA extraction. A CyQUANT cell proliferation assay was performed following manufacturer’s instructions. To evaluate fibroblasts growth rate, fluorescence was analysed 48 hours after first passage.

### Native lung fibroblasts cultured in bronchoalveolar lavage conditioned medium

Mice were sacrificed, and a bronchoalveolar lavage was immediately performed using 4 x 1 ml PBS–EDTA 0.05 mM (ref#324503 Calbiochem, Darmstadt, Germany) as previously described (13, 14). Cells were recovered by gentle manual aspiration. After centrifugation (1,200 rpm for 10 min, at 4°C), supernatant was collected and frozen at –80°C. BAL was collected from ADAM28^+/+^ and ADAM28^-/-^ mice exposed and non-exposed to OVA in a LT model of asthma. Cultured lung fibroblasts were isolated from the lungs of naïve ADAM28^+/+^ mice. Enrichment of fibroblasts culture medium was performed by adding 10% of previously collected BAL (ADAM28^+/+^ and ADAM28^-/-^ exposed to OVA or to PBS) to culture medium and cell proliferation was measured by a CyQUANT assay (Ref#C7026, Invitrogen, Merelbeke, Belgium).

### Tissue processing: RNA extraction and analyses

The right lobe of the lung previously frozen was disrupted with a Mikro-Dismembrator (Sartorius Stedim Biotech, Vilvoorde, Belgium). Total lung RNA was extracted and purified using High Pure RNA Tissue Kit (Roche ref#12033674001, Mannheim, Germany) according to the manufacturer’s instructions. Total ADAM28, membrane-bound (Variant 1, Var1) and soluble form (Variant 4, Var4) expressions were assessed by semiquantitative RT-PCR and normalized to the 28s rRNA. As 28S rRNA is the product of the precursor 45S rRNA, some portions of 45S rRNA are also amplified by the primers chosen to target 28S.

The following primers targeting respectively 28S, total ADAM28, ADAM28 Var1 and ADAM28 Var4 were used: (F) *5’- GTTCACCCACTAATAGGGAACGTGA -3’*, (R) *5’-GGATTCTGACTTAGAGGCGTTCAGT-3’*, (F) *5’-CTACTTGAGCTGCAAGTGTCCATC-3’* and (R) *5’-CAGGTCTTGCTCACAGCATTTG-3’*, (F) *5’-AGCCTCCACCTGATGTCCTAATCA-3’*, (R) *5’-TAACCCACTTTCCAGGGGTCAGTT-3’*, (F) *5’-AGCCTCCACCTGATGTCCTAATCA-3’*, and (R) *5’- cctgagggttaagagcgctagtaa-3’*. These latter primers amplifying ADAM28 variant 4 also amplify variant 5 that is a non-coding sequence.

For real-time RT-PCR, 100ng of cDNA were used, produced using RNA extracted, from whole lungs of OVA-exposed mice and control counterparts (PBS).

### Flow cytometry

To assess and quantify inflammatory cells infiltration, lungs of ADAM28^+/+^ and ADAM28^-/-^ were harvested and digested in collagenase C (1mg/ml; Gibco) prior to red blood cell lysis (Red Blood Cell Lysis Buffer, Sigma Aldrich, Saint-Louis, Missouri). Cells were stained with fluorochrome-conjugated surface antibodies during 30 minutes, fixed and permeabilized using Cytofix/Cytoperm (ref#554714 BD Biosciences, Erembodegem, Belgium) before intracellular antigen staining. Antibodies used for flow cytometry analysis were: CD5-BV421 (ref#53-7.3, BioLegend), CD4-PERCP CY 5.5 (ref#RM4-5, BD Biosciences), CD8a-BB515 (ref#53-6.7, BD Biosciences), IFN-γ-PE CY7 (ref#XMG 1.2, BD Biosciences), IL-4-APC (ref#11B11, eBioscience, Thermo Fisher Scientfic), CD45R-APC-eFluor780 (B220) (ref#RA3-6B2, eBioscience). Data were acquired on FACS CANTO II flow cytometer (BD Biosciences) and analyzed using BD FACSDiva software (BD Biosciences).

### Statistical analyses

Results are expressed as columns and scatter plots with means ± SE. Statistic comparison between groups was performed, using GraphPad InStat (GraphPad; http://www.graphpad.com). The D’Agostino Pearson and Bartlett’s tests were performed to assess normality of the values and difference of variances. Non-normal law variables were compared by nonparametric Mann Whitney test, while normal-law variables were compared using the parametric t-test. When multiple comparisons were performed a one-way ANOVA was used. Values of P<0.05 were considered as significant.

## Results

### ADAM28 expression in lungs of OVA-exposed animals

**Figure 2:**
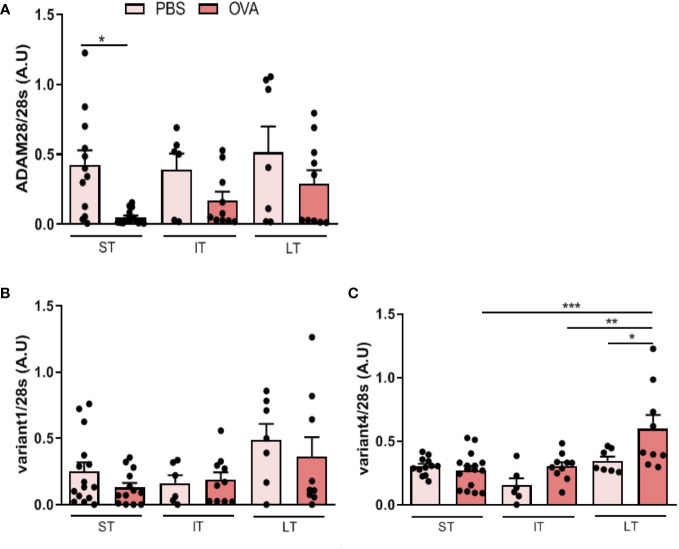
Effects of OVA exposure on ADAM28 RNA expression in the lung of mice assessed by RT-PCR on RNA extracted from lungs of mice exposed and non-exposed to OVA. Results are normalized to the 28s rRNA RT-PCR. **(A)** Quantification of relative total ADAM28 mRNA expression in lung of mice (N=2; PBS-ST n=12, OVA-ST n=15, PBS-IT n=6, OVA-IT n=10, PBS-LT n=7, OVA-LT n=10), **(B)** membrane-bound ADAM28 (Var1) mRNA expression (N=2; PBS-ST n=15, OVA-ST n=13, PBS-IT n=6, OVA-IT n=10, PBS-LT n=7, OVA-LT n=9), **(C)** secreted (Var4) ADAM28 mRNA expression (N=2; PBS-ST n=12, OVA-ST n=16, PBS-IT n=6, OVA-IT n=10, PBS-LT n=7, OVA-LT n=9). Results are represented as the mean ± SE, . *p < 0.05, **p < 0.01, ***p < 0.001 (one-way ANOVA).

In order to assess ADAM28 expression in lung in mouse models mimicking acute to chronic features of asthma, mRNAs corresponding to total ADAM28, soluble form (referred to as Variant 4: Var4) and membrane-bound form (or Variant 1: Var1) were measured by RT-PCR after ST, IT and LT allergen exposure (**Figures 2A–C**).

In the ST exposure model, total ADAM28 expression was lower in lungs of allergen-exposed mice as compared to PBS-exposed animals and ADAM28 expression correlated with the duration of exposure, i.e., animals challenged with OVA in the LT protocol had higher ADAM28 expression as compared to animals exposed to OVA in the ST protocol or in the IT protocol (**Figure 2A**). These results were confirmed by q-PCR (data not shown). Expression of the membrane-bound variant (var1) of ADAM28 was not significantly modulated at any stage of OVA exposure as compared to PBS (**Figure 2B**). On the other hand, expression of the soluble ADAM28 form (var4) was about twice higher when animals were exposed to OVA in the IT or LT protocol while not modulated in the ST protocol after OVA exposure (**Figure 2C**) and ADAM28 expression increased progressively according to the duration of allergen exposure, as it was observed for the total ADAM28.

### Effects of ADAM28 depletion on allergen-induced inflammation in lungs in long-term asthma model

**Figure 3:**
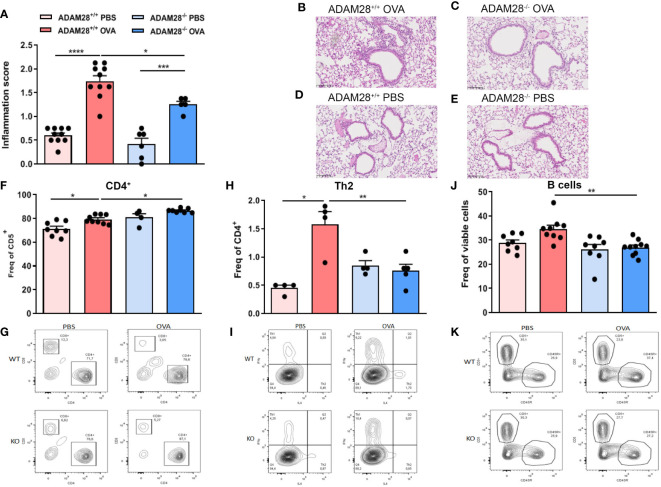
Effects of ADAM28 depletion on allergen-induced inflammation in lung tissue . Histological score estimating the peribronchial inflammation quantified from haematoxylin-eosin (H&E) stained lung section of mice lungs (N=3; ADAM28^+/+^ - PBS n=10, ADAM28^+/+^-OVA n=10, ADAM28^-/–^PBS n=6, ADAM28^-/–^OVA n=5) **(A)**. Representative H&E staining of lung sections. Scale bar: 100μm. **(B-E)**. Measurement of cell numbers in digested lung: CD4^+^/CD5^+^ ratio (N=2; ADAM28^+/+^- PBS n=8, ADAM28^+/+^-OVA n=9, ADAM28^-/–^ PBS n=4, ADAM28^-/–^OVA n=8) **(F)** and corresponding contour plot of one representative animal for each condition **(G)**; IL4^+^ and CD4^+^ cells that corresponds to the proportion of Th_2_ among Th cells (N=1; ADAM28^+/+^- PBS n=4, ADAM28^+/+^- OVA n=4, ADAM28^-/–^ PBS n=4, ADAM28^-/–^ OVA n=5) **(H)** and corresponding contour plot of one representative animal for each condition **(I)**; CD45R^+^ positive and CD5^+^ negative cells that represents proportion of B cells among lymphocytes (N=2; ADAM28^+/+^- PBS n=8, ADAM28^+/+^- OVA n=9, ADAM28^-/–^ PBS n=8, ADAM28^-/–^ OVA n=10) **(J)** and corresponding contour plot of one representative animal for each condition **(K)**. Results are represented as mean ± SE, . *p < 0.05.

In order to assess a potential role for ADAM28 in airway remodelling, ADAM28^+/+^ or ADAM28^-/-^ mice were exposed to OVA in the LT protocol. OVA-exposed animals displayed increased levels of peribronchial inflammation as compared to PBS-exposed animals regardless of the ADAM28 expression (**Figure 3A**). Nevertheless, bronchial inflammation score was significantly lower in OVA-exposed ADAM28^-/-^ as compared to OVA-exposed ADAM28^+/+^ mice. In PBS-exposed animals, levels of bronchial inflammation were low and similar in ADAM28^-/-^ and ADAM28^+/+^ (**Figures 3A–E**). ${\text{CD}}_{4}^{+}$ T lymphocytes were measured in lungs of PBS- or OVA-exposed animals by flow cytometry. ADAM28^-/-^ displayed a slightly higher recruitment of ${\text{CD}}_{4}^{+}$ T cells in the lungs upon allergen exposure as compared to ADAM28^+/+^ (**Figures 3F, G**). In the meantime, levels of ${\text{CD}}_{8}^{+}$ T lymphocytes were lower in non-exposed ADAM28^-/-^ mice as compared to the corresponding non-exposed ADAM28^+/+^(**Figure 3G**). Nevertheless, Th_2_ lymphocytes were significantly less recruited after OVA exposure in ADAM28^-/-^ animals as compared to wild-type counterparts (**Figures 3H, I**). B lymphocytes failed to increase in the lungs of ADAM28^-/-^ after OVA exposure in contrast with what was measured in ADAM28^+/+^ mice (**Figures 3J, K**).

### Measurement of lung function and airway responsiveness to methacholine after a LT allergen exposure

**Figure 4:**
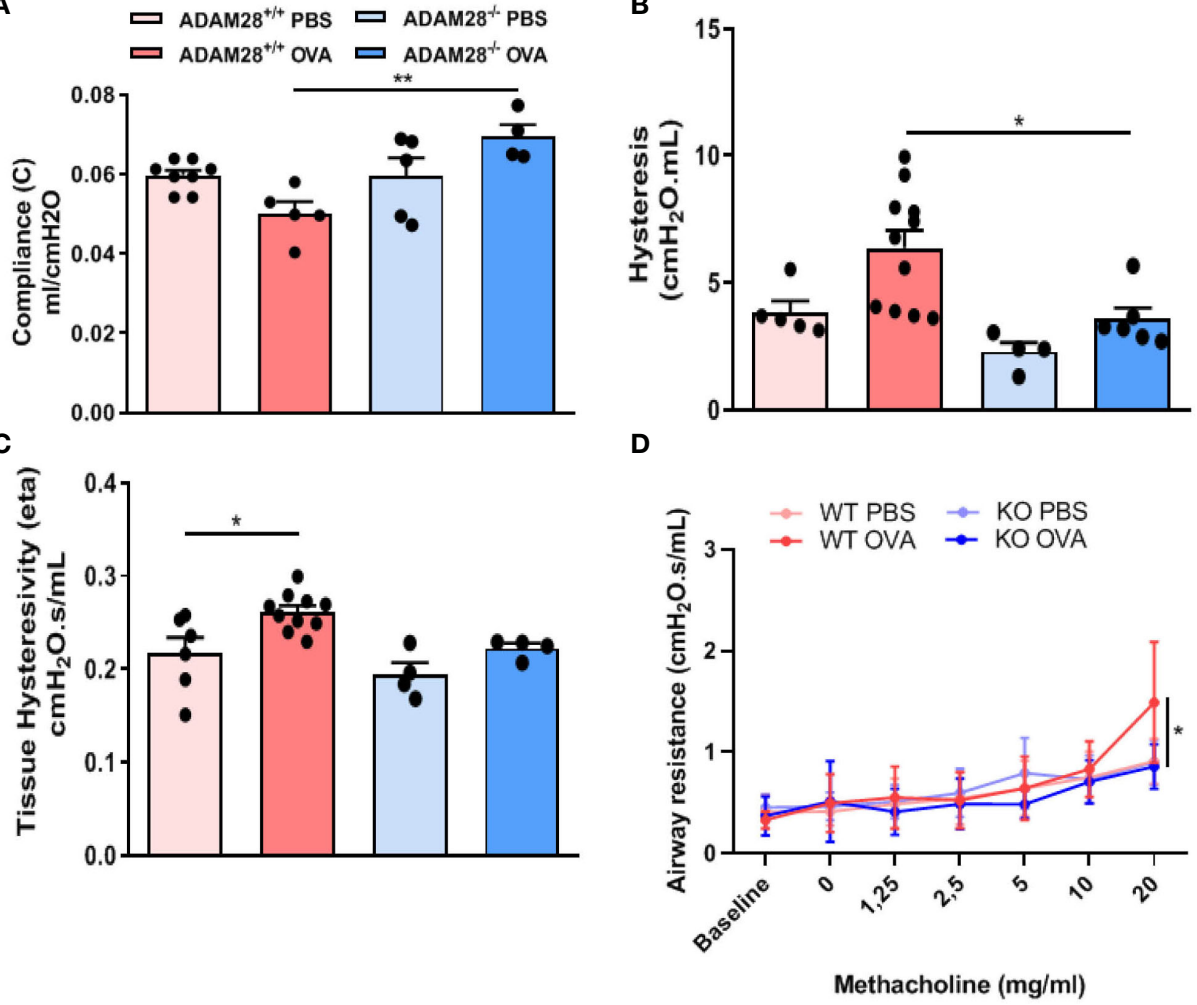
Measurement of airway function in ADAM28^-/-^ and ADAM28^+/+^ animals after allergen (OVA) or sham exposure (PBS). **(A)** Baseline lung compliance (N=2; ADAM28^+/+^- PBS n=8, ADAM28^+/+^- OVA n=5, ADAM28^-/–^ PBS n=5, ADAM28^-/–^ OVA n=4), **(B)** lung hysteresis (N=2; ADAM28^+/+^- PBS n=5, ADAM28^+/+^- OVA n=11, ADAM28^-/–^ PBS n=4, ADAM28^-/–^ OVA n=6) **(C)** lung hysteresivity (N=2; ADAM28^+/+^- PBS n=6, ADAM28^+/+^- OVA n=10, ADAM28^-/–^ PBS n=4, ADAM28^-/–^ OVA n=4). **(D)** Measurement of the airway responsiveness. The airway resistance was measured *via* the forced oscillation technique (Flexivent^®^) 24h after the last OVA or PBS exposure after exposure to increasing concentrations of methacholine. Results are expressed as mean ± SE, (N=2; ADAM28^+/+^- PBS n=7, ADAM28^+/+^- OVA n=10, ADAM28^-/–^ PBS n=3, ADAM28^-/–^ OVA n=6). *p < 0.05, **p < 0.01 (One way ANOVA).

At baseline, i.e., in PBS-exposed mice, there were no significant differences between ADAM28^-/-^ and ADAM28^+/+^ animals regarding lung function parameters measured by Flexivent^®^ (compliance (C), hysteresis and airway resistances) (**Figures 4A–C**). In the LT protocol, OVA-exposed ADAM28^+/+^ mice displayed changes in lung function (significant decrease of compliance, and increase of hysteresis and airway resistances) when compared to PBS-exposed mice. In contrast, lung function measurements in the LT protocol (evaluated by compliance, hysteresis and airway resistances measurements) in ADAM28^-/-^ were not modified after OVA exposure and remained similar to the values obtained from sham-exposed mice.

Bronchial responsiveness following exposure to increasing doses of methacholine was measured using the FlexiVent^®^ system in ADAM28^+/+^ or ADAM28^-/-^ mice exposed for 90 days to OVA or PBS. During methacholine challenge, OVA-exposed ADAM28^+/+^ animals displayed a classical increase in airway resistances when compared with PBS exposed counterparts while, in sharp contrast, OVA-exposed ADAM28^-/-^ animals did not reach similar levels of airway resistance after being exposed to the highest doses of methacholine and displayed values similar to PBS-exposed animals (**Figure 4D**).

### Allergen-exposed ADAM28^-/-^ mice display significantly less features of airway remodelling in LT asthma model

**Figure 5:**
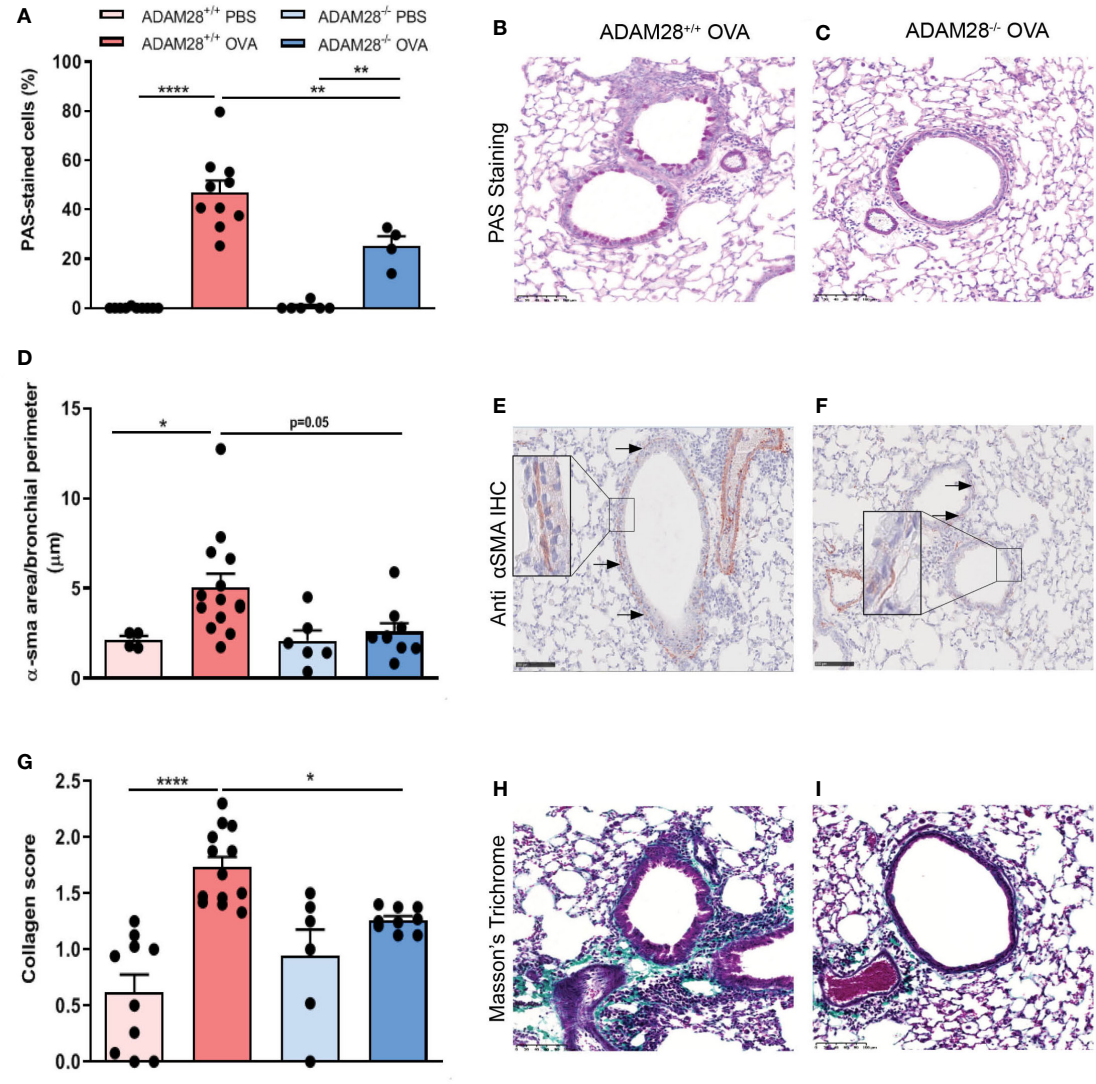
Assessment of airway remodelling in the lung of ADAM28^-/-^ and ADAM28^+/+^ mice exposed to allergens for 90 days (LT protocol). **(A)** Quantification of the percentage of PAS-positive epithelial cells per bronchi (N=2; ADAM28^+/+^- PBS n=10, ADAM28^+/+^- OVA n=10, ADAM28^-/–^ PBS n=6, ADAM28^-/–^ OVA n=5). **(B, C)** Representative PAS staining of lung sections of ADAM28^+/+^ and ADAM28^-/-^ mice exposed to OVA. Scale bar: 100 μm. **(D)** Assessment of smooth muscle area around the bronchi stained by IHC against α-SMA reported to perimeter of the epithelial basement membrane (mean of 7 bronchi per mouse) (N=2; ADAM28^+/+^- PBS n=4, ADAM28^+/+^- OVA n=14, ADAM28^-/–^ PBS n=6, ADAM28^-/–^ OVA n=9). **(E, F)** Representative IHC with an anti-α-SMA in lung sections of ADAM28^+/+^ and ADAM28^-/-^ mice exposed to OVA. Scale bar: 100 μm. **(G)**. Collagen deposition score related to the thickness collagen (mean of 7 bronchi randomly selected per mouse, each one scored from 0 to 3) (N=2; ADAM28^+/+^- PBS n=10, ADAM28^+/+^- OVA n=13, ADAM28^-/–^ PBS n=6, ADAM28^-/–^ OVA n=9). **(H, I)** Collagen stained by Masson’s Trichrome staining of lung sections of ADAM28^+/+^ and ADAM28^-/-^ mice exposed to OVA. Scale bar: 100 μm. Results are represented as the mean ± SE. *p < 0.05, **p < 0.01, ***p < 0.001 (One way ANOVA).

Airway remodelling was evaluated in the LT protocol after allergen (OVA) or PBS exposure by histology and immuno-histological analysis. Mucous hyperplasia was measured by Periodic Acid-Schiff (PAS) staining. Percentages of mucous cells in the airway epithelium were higher in OVA-exposed mice as compared to PBS-exposed counterparts (**Figures 5A–C**). Percentages of mucous cells after OVA exposure were significantly lower in ADAM28^-/-^ as compared to ADAM28^+/+^ animals (**Figures 5A–C**). Airway smooth muscle hyperplasia was quantified by the measurement of alpha-smooth muscle actin (α-sma) in immunohistochemistry (**Figures 5D–F**). In OVA-exposed ADAM28^+/+^ mice, the area occupied by α-sma was significantly increased in airway walls as compared to PBS counterparts while this area did not increase in ADAM28^-/-^ exposed to allergens (**Figures 5D–F**). ADAM28^+/+^ mice exposed to allergens displayed a significantly increased collagen deposition in the airway walls as measured by Masson’s trichrome staining. In contrast, the extent of collagen deposition in the airway walls was significantly lower in ADAM28^-/-^ exposed to allergens (**Figures 5G–I**).

### Lung fibroblasts express ADAM28 mRNA and their proliferation rate is lower after OVA exposure when they originate from ADAM28^-/-^

**Figure 6:**
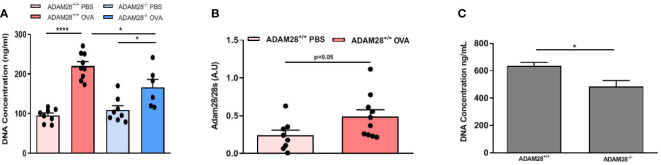
Study of lung fibroblasts. **(A)** CyQuant measurement of cell proliferation of fibroblasts extracted from lungs of ADAM28^-/-^ and ADAM28^+/+^ mice exposed to OVA or PBS. DNA levels were measured in cell culture during 48h (N=2; ADAM28^+/+^- PBS n=8, ADAM28^+/+^- OVA n=9, ADAM28^-/–^ PBS n=8, ADAM28^-/–^ OVA n=6). **(B)** RT-PCR measurement of ADAM28 mRNA expression in fibroblasts extracted from wild-type mice exposed to OVA or PBS for 90 days (N=2; ADAM28^+/+^- PBS n=8, ADAM28^+/+^- OVA n=10). **(C)** Measurement by CyQuant of cell proliferation of fibroblasts extracted from lungs of naive mice exposed during 48 hours to media enriched with 10% of BALF from ADAM28^+/+^ or ADAM28^-/-^ mice exposed to OVA in the LT model. DNA levels were measured in cell culture during 48h (N=1; ADAM28^+/+^- OVA n=7, ADAM28^-/–^ OVA n=7). Results are represented as the mean ± SE. *p < 0.05, ***p < 0.001 (**Figure 6A**: One way ANOVA, **Figures 6B, C**: Mann-Withney).

In order to study the potential role of ADAM28 in the modulation of airway remodelling, fibroblasts were isolated from lungs of OVA- or PBS-exposed animals (LT protocol) and cultured in order to evaluate their ability to produce ADAM28 mRNA and to proliferate. Proliferation rate of freshly isolated fibroblasts was evaluated after the first passage by measuring DNA levels in cell culture during 48h by Cyquant analysis. Fibroblast proliferation was significantly increased after OVA exposure, regardless of their extraction from ADAM28^+/+^ or ADAM28^-/-^ mouse lungs (**Figure 6A**). However, the proliferation of fibroblasts extracted from lungs of OVA-exposed ADAM28^-/-^ animals was significantly lower as compared with OVA-exposed ADAM28^+/+^ (**Figure 6A**). No significant difference was found regarding ADAM28 mRNA expression measured by RT-PCR in fibroblasts extracted from OVA- or PBS-exposed wild-type mice (**Figure 6B**).

### Bronchoalveolar lavage from OVA-exposed ADAM28^-/-^ fail to stimulate fibroblast proliferation

Lung fibroblasts were isolated from naïve ADAM28^+/+^ mice and cultured with a medium containing 10% BAL. Cell proliferation was evaluated by measuring DNA levels using CyQUANT during 48 hours. Fibroblast proliferation was significantly increased when cells were cultured with BAL from OVA-exposed ADAM28^+/+^ (LT model) in contrast to what was observed when fibroblasts were incubated with BAL collected from OVA-exposed ADAM28^-/-^ (**Figure 6C**).

## Discussion

We report here that total ADAM28 expression in lungs is lower after allergen exposure in a mouse model of asthma. However, the expression of the secreted variant of ADAM28 (var4) gradually increase with the duration of allergen exposure with levels that stay lower in allergen-exposed animals as compared to sham-exposed mice. As our working hypothesis was that ADAM28 could play a role in asthma-related airway remodelling, we focused on a long term allergen-exposure model (LT) obtained after 90 days of exposure to allergens where the highest levels of ADAM28 expression in the lung tissue were measured.

Although its precise biological functions are still uncertain, ADAM28 might play a role in key mechanisms leading to asthma-related inflammation and airway remodelling. We show indeed that after OVA exposure, ADAM28^-/-^ mice does not display the same level of inflammatory cells recruitment in the peribronchial area as observed in ADAM28^+/+^ mice. Interestingly, Th_2_ cell numbers fail to increase after allergen exposure in the lungs of ADAM28^-/-^ suggesting a profound dysregulation of immunological pathways when these ADAM28 deficient mice are exposed to allergens. This is in line with the lower levels of CD8 cells reported earlier in cancer models in these mice (20). Together with lower levels of inflammation in ADAM28^-/-^ animals, key features of airway remodelling (glandular hyperplasia, smooth muscle cell hyperplasia and collagen deposition) are significantly smaller after allergen exposure as compared to wild-type animals.

As we hypothesized that ADAM28 could cleave membrane-bound or soluble mediators and activate these molecules, we planned to assess the possible biological effects on fibroblasts (that could contribute to explain our findings regarding airway remodeling). One of the mechanisms that might account for these differences is an inhibition of fibroblast proliferation as shown in this article on fibroblasts extracted from lungs of ADAM28^-/-^ and wild-type counterparts. As fibroblasts incubated with the BAL from ADAM28^-/-^ mice also displayed a lower proliferation rate, this suggests that a soluble factor in the BAL can stimulate fibroblast proliferation and is not produced nor activated in the absence of ADAM28. We chose to study effects of the BAL fluid since it contains a large number of mediators that have potentially been processed by ADAM28. However, the identification of such factor that might be a substrate for ADAM28 is not achieved yet. Specific techniques as iTRAQ-TAILS followed by mass spectrometry might be helpful to answer that question by unveiling cleavages performed by membrane-bound proteases (23).

In line with a significantly lower remodelling of the airways of ADAM28^-/-^ animals after OVA challenge, we measured significantly lower airway responsiveness in these animals as compared to wild-type mice after allergen exposure. A possible protection of ADAM28^-/-^ mice against structural changes of the airways is plausible since we measured significantly lower compliance, an increase of hysteresis and hysteresivity (η) in ADAM28^+/+^ exposed to allergens. These parameters are closely related to the remodelling classically observed in models of asthma after 90 days allergen exposure therefore confirming our histological observations (lower airway remodelling in ADAM28^-/-^ mice as compared to ADAM28^+/+^ after OVA exposure).

The exact mechanisms linking ADAM28 and a modulation of airway remodelling and responsiveness in asthma are still to be unveiled but there are arguments to hypothesize that ADAM28 contributes to the events leading to the asthma phenotype. The ability of this protease to cleave the low affinity IgE receptor CD_23_ present at the surface of B-cells, monocytes, macrophages and eosinophils (24, 25) could interfere with different key processes. Indeed, this cleavage of CD_23_ generates a soluble form of CD_23_ as already demonstrated for other ADAM proteases including ADAM8 (26, 27). However, this effect on CD_23_ could be negligible since ADAM10 was recognized as the main sheddase for CD 23 (28). Nevertheless, control of CD23 is of key importance in mechanisms of asthma and allergy as this mediator modulates T cell activation (29, 30). As ADAM28 has the capacity to bind various integrins, it can also be hypothesized that this protease might interfere with inflammatory and stromal cells trafficking (17). For example, ADAM28 recognizes α_9_β_1_ as well as α_4_β_1_ which contribute to adhesion and transendothelial migration of neutrophils and lymphocytes, respectively (31–34). Also, it was shown that soluble ADAM28 is able to enhance α4β1-dependent cell adhesion to VCAM-1 (vascular cell adhesion molecule-1) therefore influencing lymphocyte adhesion to endothelium and trafficking across the capillary walls (17). This is possibly the cause of the lack of migration of Th_2_ lymphocytes to the lung after allergen exposure in ADAM28^-/-^ animals reported in this work.

We recently reported that ADAM28 depletion in mice causes increased tumour cell dissemination in lungs by decreasing the cancer cytotoxicity mediated by CD_8_ lymphocytes (20). However, ADAM28 expression has been reported in thymic epithelial cells suggesting a role in T lymphocyte differentiation (32–35) but mice depleted for ADAM28 did not display any abnormalities in thymocytes and T lymphocytes differentiation (20). A drastic reduction of CD8^+^ T cells was reported in spleen of ADAM28^-/-^ (20). In this study, we report that CD8 lymphocytes are lower in ADAM28^-/-^ as compared to ADAM28^+/+^ animals. The role of CD8^+^ lymphocytes in asthma is complex and has been nicely reviewed by Lourenço et al. (36). Inhibition studies using depleting antibodies suggest that ${\text{CD}}_{4}^{+}$ lymphocytes are not *per se* sufficient to induce airway remodelling (37) suggesting a role for other actors as ${\text{CD}}_{8}^{+}$ cells. These cells are heterogeneous and a subset of ${\text{CD}}_{8}^{+}$ cells was reported to produce IL13 and was associated with airway obstruction suggesting a plausible role of these cells in airway remodelling (38). Moreover, IL13 plays a significant role in airway remodelling and was shown to increase the pro-fibrotic gene expression in fibroblasts (39).

Interestingly, ADAM28 might activate pathways leading to or supporting asthma-related inflammation by modulating the biological activity of mediators as TNF-α. Indeed, ADAM28 might activate TNF-α acting as a sheddase able to release mature TNF-α in the supernatant of ADAM28 transfected HEK-293 cells (40). Also, inhibition of endogenous ADAM28 in macrophages resulted in a reduced mature TNF-α release (40). Recently, it was suggested that ADAM28 might play a role in key immunomodulatory mechanisms since CD20^+^/CD22^+^/ADAM28^+^ B cells were shown to promote response to immune checkpoint inhibitor therapy in non-small-cell lung cancer (41).

Regarding the differences of expression of ADAM28 Var4 in the mouse model, the primers used to amplify Var4 also recognize and amplify Var5 that is a non-coding sequence. However, we cannot rule out a possible interference with measurements of RNA corresponding to Var4.

Full ADAM28 depletion in mouse does not lead to any spontaneous phenotype and specifically no developmental abnormalities in bronchial tree, alveolae architecture, or bronchial epithelium (20). As previously reported for many other proteases, the stimulation of inflammation is able to unveil specific functions of ADAM28. Furthermore, our observations suggest that ADAM28 contributes to mechanisms leading to asthma-related airway inflammation and remodelling but it is probably not the final effector and further experiments are needed to fully unveil the precise molecular mechanism.

## Data availability statement

The raw data supporting the conclusions of this article will be made available by the authors, without undue reservation.

## Ethics statement

The animal study was reviewed and approved by Comité d’éthique animal hospitalo-universitaire - Université de Liège et CHU Liège.

## Author contributions

GB, CH, FP, AG, MJN, OC, CG performed the bench work and experiments described in the manuscript. GB drafted the figures. FP, DC, AG, and MJN edited the final version of figures. AN, PL, NR supervised the experiments and contributed to the interpretation of results and to manuscript redaction. NR created the ADAM28-/- mice. DC started the research program, applied for grants to fund the research program, supervised the bench work and the interpretation of results, finalized the manuscript and submitted the manuscript and the revised version of the manuscript. All authors contributed to the article and approved the submitted version.

## Supplementary material

Supplementary Figure 1 Gating strategy for flow cytometry: Identification of IL4 and IFNγ production by CD4+ T cells. Debris and doublets were excluded based on FSC and SSC. B lymphocytes were defined as live cells positive for CD45R and negative for CD5. CD4+ T cells were identified as live CD5+ CD4+ cells. IL4 and IFNγ was then assessed in this population. CD8+ T cells were identified as alive CD5+ CD8+ cells.
