# Supplementary figures and images for: Role for the metalloproteinase ADAM28 in the control of airway inflammation, remodelling and responsiveness in asthma
Source: Front Immunol. 2023 Jan 5;13:1067779. doi: 10.3389/fimmu.2022.1067779 (PMC9851272; doi:10.3389/fimmu.2022.1067779)

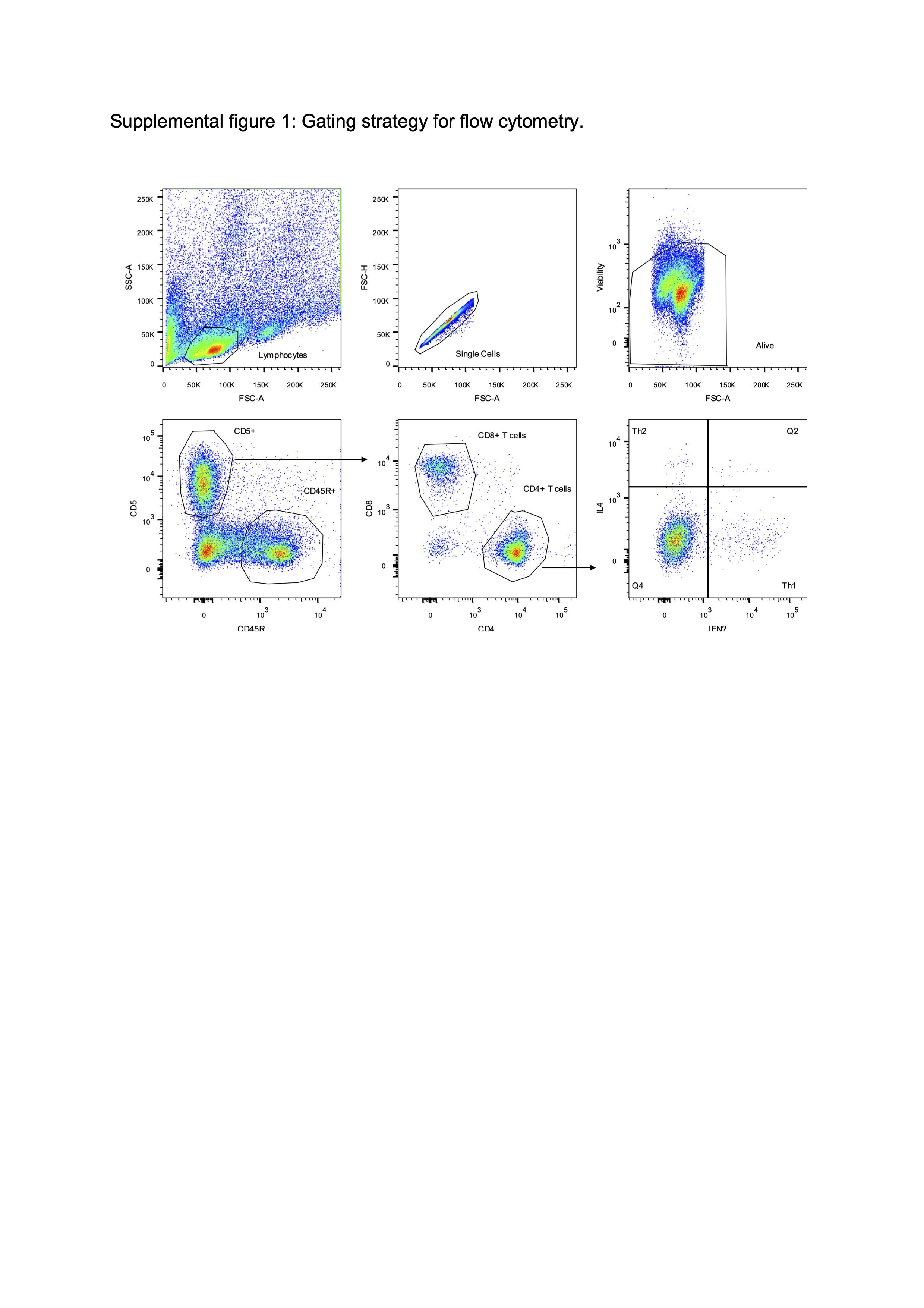

Supplement: Supplementary Figure 1 — Gating strategy for flow cytometry: Identification of IL4 and IFNγ production by CD4+ T cells. Debris and doublets were excluded based on FSC and SSC. B lymphocytes were defined as live cells positive for CD45R and negative for CD5. CD4+ T cells were identified as live CD5+ CD4+ cells. IL4 and IFNγ was then assessed in this population. CD8+ T cells were identified as alive CD5+ CD8+ cells. [file Image_1.jpeg]
